# A smaller heart-aorta-angle associates with ascending aortic dilatation and increases wall shear stress

**DOI:** 10.1007/s00330-020-06852-3

**Published:** 2020-04-22

**Authors:** S. Petteri Kauhanen, Timo Liimatainen, Elina Kariniemi, Miika Korhonen, Johannes Parkkonen, Juska Vienonen, Ritva Vanninen, Marja Hedman

**Affiliations:** 1 Doctoral Programme of Clinical Research, University of Eastern Finland, Kuopio, Finland; 2 Department of Clinical Radiology, Clinical Imaging Center, Kuopio University Hospital, Kuopio, Finland; 3 Research Unit of Medical Imaging, Physics and Technology, University of Oulu, Oulu, Finland; 4 Department of Diagnostic Radiology, Oulu University Hospital, Oulu, Finland; 5 Department of Clinical Physiology and Nuclear Medicine, Clinical Imaging Center, Kuopio University Hospital, Kuopio, Finland; 6 School of Medicine, Clinical Radiology, University of Eastern Finland, Kuopio, Finland; 7 Department of Cardiothoracic Surgery, Heart Center, Kuopio University Hospital, Kuopio, Finland

**Keywords:** Aorta thoracic, Aortic aneurysm, Heart ventricles, Tomography x-ray computed, Magnetic resonance imaging

## Abstract

**Objectives:**

The aim of this study was to evaluate whether the orientation of the heart, measured as an angle between the long axis of the heart and ascending aorta midline (heart-aorta-angle, HAA), associates with ascending aortic (AA) dilatation. Furthermore, the association between HAA and wall shear stress (WSS) was studied.

**Methods:**

HAA was retrospectively measured in 1000 consecutive coronary artery computed tomographic angiography (CCTA) images in patients with low-to-moderate pretest probability for coronary artery disease (CAD). To evaluate the effects of HAA on AA flow, 4D flow MRI was performed for 28 patients with AA dilatation (> 40 mm) and WSS was analyzed.

**Results:**

The mean age of patients undergoing CCTA was 52.9 ± 9.8 years; 66.5% were women. Their median HAA was 128.7° and interquartile range 123.3–134.1°. HAA was significantly smaller in patients with dilated AA (median 126.7° [121.3–130.8°]) compared with the patients with normal AA (median 129.5° [124.3–135.3°], *p* < 0.001). HAA was smaller in males (*p* < 0.001) and in patients with diabetes (*p* = 0.016), hypertension (*p* = 0.001), CAD (*p* = 0.003), hypercholesterolemia (*p* < 0.001), and bicuspid aortic valve (*p* = 0.025) than without these factors. In a subpopulation without any of these underlying diseases (*n* = 233), HAA was still significantly smaller in the patients with dilated AA (median 127.9° [124.3–134.3°]) compared with patients with normal AA (median 131.9° [127.6–136.9°], *p* = 0.013). In 4D flow MRI, a smaller HAA correlated with increased total WSS in the outer curvature of the proximal AA (*r* = − 0.510, *p* = 0.006).

**Conclusion:**

A smaller HAA associates with AA dilatation and affects the blood flow in the proximal AA.

**Key Points:**

*• A smaller angle between the long axis of the heart and ascending aorta midline associated with ascending aortic dilatation.*

*• A smaller heart-aorta-angle correlated with increased total wall shear stress in the outer curvature of the proximal ascending aorta.*

## Introduction

The diameter of thoracic aorta has been shown to increase with aging, male gender, and increased body surface area (BSA) [1]. Furthermore, ascending aortic (AA) dilatation is associated with conventional cardiovascular risk factors, such as hypertension and smoking [2–4]. Also, the length of the thoracic aorta has been shown to be related to age; i.e., aortic elongation may be a part of the normal aging process. Adriaans et al have demonstrated that the thoracic aortic length increases by 59–66 mm between the ages of 20 and 80 years [5].

However, the associations between AA dilatation and the angle between the heart and AA (the heart-aorta-angle, HAA) have not been studied previously. Under normal conditions, the heart is oriented with the right ventricle on the anterior side and the left atrium on the posterior side [6]. The axis of the heart is orientated at approximately 45° to the left of an anteroposterior line drawn from the spine to the anterior chest wall [7]. It has been described that the heart is oriented vertically downwards in the “Valentine” position, which means that the heart is a solitary organ and provides no reference point for its location within the chest [8]. In healthy subjects, the HAA has been reported to be approximately 140 ± 7° [9].

In patients with AA dilatation, the aortic blood flow has been shown to be displaced even in the case of normal tricuspid aortic valve [10]. Furthermore, the displaced flow may lead to increased wall shear stress (WSS) on the displaced side of the AA [10]. A bicuspid aortic valve (BAV) and aortic stenosis have also been shown to associate with increased WSS values [11].

The purposes of this study were (1) to investigate the association between HAA and AA dilatation and (2) to analyze whether the HAA has an influence on the blood flow and WSS in the AA.

## Methods

The study was approved by the Ethics Committee, Hospital District of Northern Savo. Coronary artery computed tomography angiography (CCTA) imaging was performed on the basis of clinical indications; thus, the patients were not exposed to additional radiation dose. The patients’ clinical treatment was unaffected by the study. The populations of the present study have also been analyzed in the prior publications [10, 12, 13].

### Patient population

#### CCTA patient population

This retrospective study examined 1065 consecutive patients with low-to-moderate pretest probability for coronary artery disease (CAD) and without pre-existing aortic disease scheduled for CCTA in Kuopio University Hospital between January 2012 and March 2018. Sixty-four patients were excluded due to motion artifacts or inadequate visibility of AA in CCTA images and one patient who was aged under 16 years. The mean age of the CCTA study population (*n* = 1000) was 52.9 ± 9.8 years and the majority of the patients were women (*n* = 665, 66.5%). Patients’ baseline characteristics are presented in Table 1.

**Table 1:** Baseline characteristics of the CCTA and 4D flow MRI population. *BSA*, body surface area; *CAD*, coronary artery disease; *CCTA*, coronary computed tomography angiography

	CCTA population			4D flow MRI population		
	All patients, *n* = 1000	Males, *n* = 335	Females, *n* = 665	All patients, *n* = 28	Males, *n* = 25	Females, *n* = 3
Age (years)	52.9 ± 9.8	48.5 ± 10.8	55.1 ± 8.5	65.6 ± 6.3	65.3 ± 6.5	68.3 ± 3.8
Height (cm)	168.7 ± 9.6	178.3 ± 6.3	163.6 ± 6.6	177.6 ± 7.1	179.2 ± 5.4	164.3 ± 6.0
Weight (kg)	80.1 ± 17.7	90.7 ± 16.1	74.3 ± 15.8	91.7 ± 16.6	94.6 ± 14.9	69.0 ± 12.1
BSA (m^2^)	1.9 ± 0.2	2.1 ± 0.2	1.8 ± 0.2	2.1 ± 0.2	2.2 ± 0.2	1.8 ± 0.2
Diabetes	80 (8.0)	30 (9.0)	50 (7.5)	5 (17.9)	4 (16.0)	1 (33.3)
Hypertension	445 (45.5)	143 (42.7)	312 (46.9)	23 (82.1)	20 (80.0)	3 (100.0)
Hypercholesterolemia	500 (50.0)	160 (47.8)	340 (51.1)	12 (42.9)	10 (40.0)	2 (66.7)
Positive family history for CAD	572 (57.2)	168 (50.1)	404 (60.8)	3 (10.7)	2 (8.0)	1 (33.3)
Smoking	254 (25.4)	123 (36.7)	131 (19.7)	3 (10.7)	3 (12.0)	0
Normal CCTA	625 (62.5)	180 (53.7)	445 (66.9)	–	–	–
Over 50% stenosis in CCTA	149 (14.9)	55 (16.4)	94 (14.1)	–	–	–
Coronary calcification in CCTA	226 (22.6)	100 (29.9)	126 (18.9)	–	–	–
Bicuspid aortic valve	31 (3.1)	22 (6.6)	9 (1.4)	0	0	0
Mechanical aortic valve	1 (0.1)	0	1 (0.2)	0	0	0

#### 4D flow MRI patient population

The power calculation was performed for the Spearman test with power 0.8 and obtained *p* value and significance level 0.05. Based on the results of power calculation, this prospective study included 28 patients with AA dilatation who were imaged with aortic magnetic resonance imaging (MRI) combined with 4D flow analysis between August 2017 and December 2019 in Kuopio University Hospital and who had prior thoracic CT scans. All patients had normal tricuspid anatomy of the aortic valve without aortic stenosis. Aortic dimensions were measured from the MRI images and the previously performed thoracic CT scans were used for the measurement of HAA. The mean age of 4D flow MRI population (*n* = 28) was 65.6 ± 6.3 years and 25 (89.3%) of patients were male. Patients’ baseline characteristics are presented in Table 1.

### CCTA imaging

CCTA imaging was performed during mid-diastole according to the routine clinical practice using four different CT scanners capable of ECG-gated fast coronary CT imaging (Somatom Definition AS 64; Somatom Definition AS+ 128; Definition Edge; and Definition Flash, Siemens Medical Solutions). Collimation was 64 × 0.6 mm with the Somatom Definition AS 64 and 128 × 0.6 mm for the other scanners. The specific imaging procedure has been presented in a previous study [12].

### Magnetic resonance imaging

MRI was performed with a Siemens Magnetom Aera 1.5-T scanner. MRI angiography was performed without contrast media with true fast imaging with steady-state precession (TRUFI) with a respiratory navigator. 4D flow MRI was performed with ECG-gating and free-breathing without contrast media. The imaging parameters were selected in line with the 4D flow consensus statement [14]. The detailed imaging procedure has been presented in a previous study [10].

### Data assessment

One experienced observer (S.P.K.) retrospectively analyzed the CCTA images on an IDS7 diagnostic workstation (version 17.3.6; Sectra Imtec). The AA was divided into 3 planes: sinus valsalva, sinotubular junction, and mid-AA. According to the current international recommendations, aortic diameters were measured from the outer-to-outer vascular wall perpendicular to the centerline of the vessel [1]. Aortic valve anatomy (tricuspid, bicuspid, or mechanical aortic valve prosthesis), middle diastolic diameter of cardiac left ventricle (LV), area of left atrium, thickness of left ventricular posterior wall, and interventricular septum were registered. The thickness of intraventricular septum was further dichotomized as under the mean value and over the mean value.

**Fig. 1:**
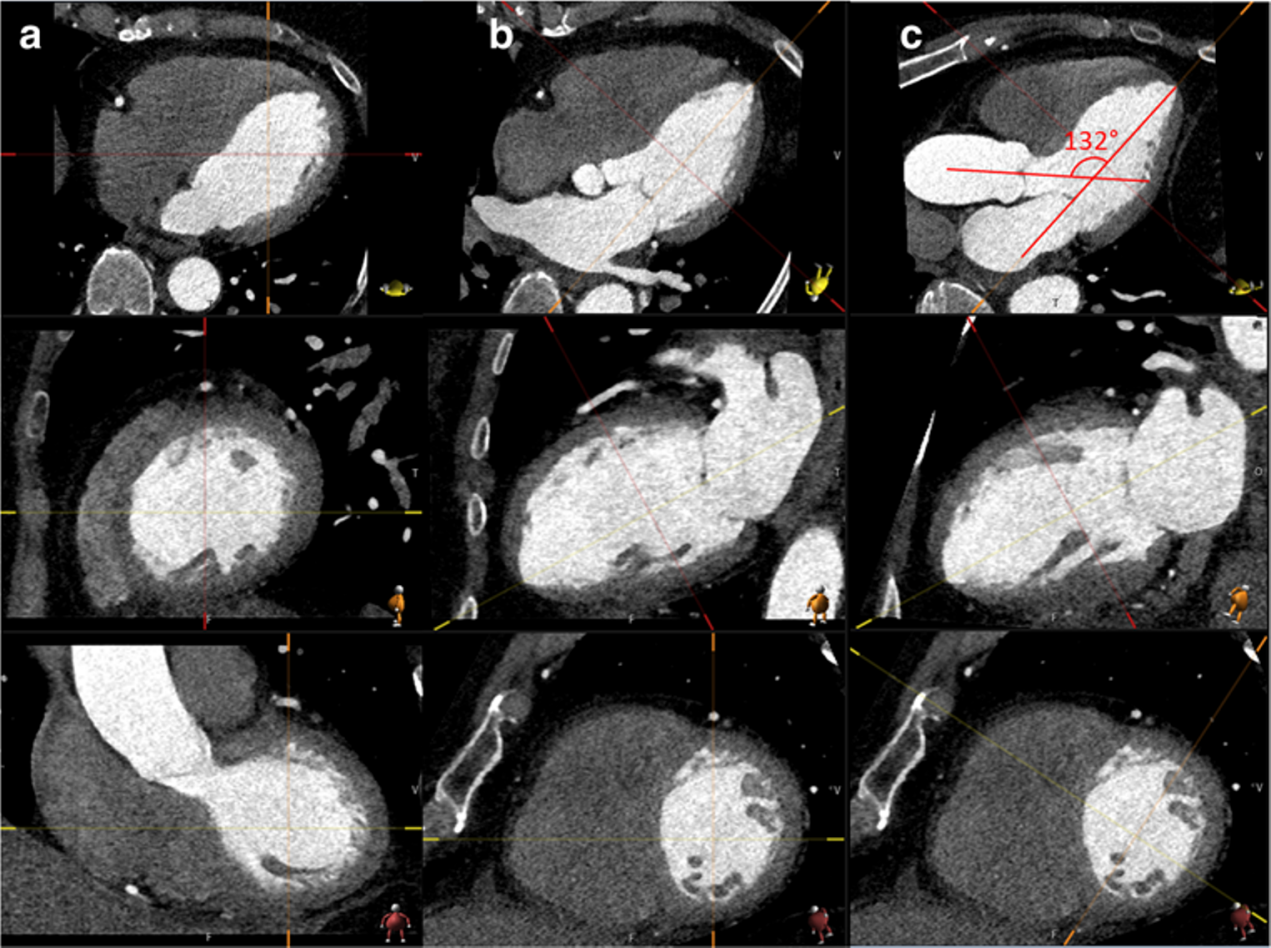
Multiplanar reformatted images of the heart-aorta-angle (HAA) measurement. An axial direction on the top, a sagittal direction on the middle, and coronal direction on the bottom lines. **a** The first step was to set the middle of the left ventricle in coronal and sagittal images. **b** The second step was to set the longitudinal line through the center of the mitral valve annulus and left ventricular apex in axial and sagittal images. **c** The last step was to take the 3-chamber projection by turning the short axis line anticlockwise in coronal images so that AA become present and measured the angle between the left ventricle long axis line and middle AA line. The HAA was 132° in this case. The HAA was measured according to the previous report [9]

The HAA was measured in multiplanar reformatted images as described earlier in the literature [9]. The 3-chamber projection was used to draw the midline through the LV from the apex through the center of the mitral valve and the midline of the left outflow tract and the AA. The HAA measurement method is presented in Fig. 1. To assess interobserver reproducibility, two observers (S.P.K. and E.K.) independently measured the HAA from 100 CCTA images. To assess the intraobserver reproducibility, one observer (E.K.) repeated the 100 HAA measurements.

#### AA dilatation classification methods

Main classification method for aortic dilatation was based on the current (2014) European Society of Cardiology (ESC) guidelines. According to these guidelines, the AA is considered dilated regardless of the gender when its greatest diameter exceeds 40 mm in any of the three measurement planes [1].

Hannuksela et al proposed age-related formula (upper limit for normal mid-AA = 30 mm + 0.20 × age) and body size–adjusted formula, which were selected as additional classification methods [13, 15]. To assess the body size adjusted formula, aortic height index (AHI) was calculated ($\mathrm{AHI}=\frac{\mathrm{Aortic}\ \mathrm{diameter}\ \left(\mathrm{m}\mathrm{m}\right)}{\mathrm{patient}\ \mathrm{height}\ \left(\mathrm{m}\right)}\Big)$. The upper limit for normal mid-AA was set as 23.3 mm/m according to our prior publication [13].

#### MRI flow parameters

**Fig. 2:**
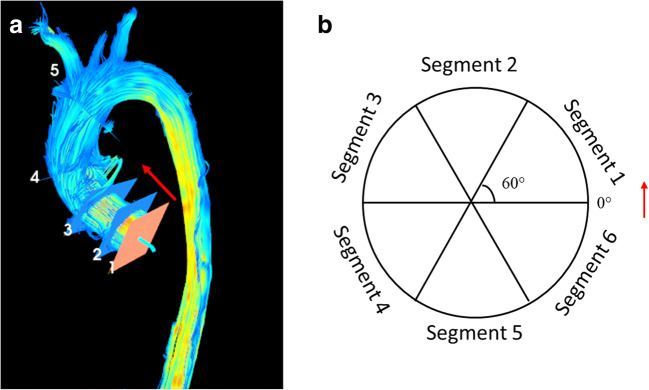
**a** Wall shear stress was analyzed in 5 planes of ascending aorta: (1) sinus valsalva, (2) sinotubular junction, (3) proximal part of tubular aorta, (4) mid-ascending aorta, and (5) proximal part of the aortic arch. **b** The aortic ring was divided into six segments (each 60°). The red arrow points to the inner curvature of AA indicating the zero point of the WSS measurements

Circumferential wall shear stress (WSS_C,_ parallel to the emitter plane), axial (WSS_A_, perpendicular to emitter plane), and total WSS (WSS_T_, geometric sum of WSS_C_ and WSS_A_) were measured at 5 different levels of AA (Fig. 2): (1) sinus valsalva, (2) sinotubular junction, (3) proximal tubular part, (4) mid-AA, and (5) proximal part of the aortic arch. The aortic ring was divided into six 60° segments. The starting point (0°) was defined to be in the inner curve of AA and segment 1 covering from 0° to 60° in a counter-clockwise direction [10]. Peak systolic WSS was obtained for the analysis. Intra- and interobserver reproducibility analyses for the flow parameters of the 4D flow have been previously published and proved to be moderate [10].

#### Risk factors

Risk factors for cardiovascular diseases as well as other baseline characteristics were collected from the medical records. The patient was defined as hypertensive if he/she was receiving medication for hypertension and as diabetic and if thepatient had two separate fasting plasma glucose levels ≥ 7.0 mmol/l or ≥ 11 mmol/l in a glucose tolerance test or HbA_1C_ ≥ 48 mmol/l. Current smokers and those who had stopped continuous smoking less than 30 years previously were considered smokers. Based on the coronary artery findings in the CCTA, the patients were dichotomized as positive or negative in terms of CAD. Hypercholesterolemia was determined according to the Finnish national recommendations as high LDL (> 3 mmol/l) and low HDL (males < 1 mmol/l, females < 1.2 mmol/l) concentrations as described previously [12]. Body mass index was dichotomized as normal weight (< 25 kg/m^2^) or as overweight or obesity (≥ 25 kg/ m^2^) [16]. Age was dichotomized as under the mean value or over the mean value.

### Statistical analysis

The normality of the HAA data was analyzed using the Kolmogorov-Smirnov test. Skewed distributed parameters were tested with the Mann-Whitney *U* test, the results being presented as median and interquartile range (IQR). Correlations between the HAA and continuous scaled parameters were tested by using the Spearman correlation test. Multivariate logistic regression was used to test the association between HAA and different classification methods of AA dilatation.

Paired samples *t* test was used to test the systematic error in intra-and interobserver analyses. Intraclass correlation coefficients (ICCs) using a two-way mixed effects model with absolute agreement were used to calculate intra- and interobserver reproducibility. ICC values from 0.0 to 0.2 were considered negligible, from 0.2 to 0.4 very low, from 0.4 to 0.7 moderate, from 0.7 to 0.9 strong, and from 0.9 to 1.0 very strong.

Statistical significance was set to *p* < 0.05 and high statistical significance to *p* < 0.001. All statistical analyses were performed using the IBM SPSS Statistics 25. Statistical analysis was performed in collaboration with a biostatistician.

## Results

According to the ESC guidelines, 230 patients (23%) were stratified as having AA dilatation in the CCTA population when the measurement results from all three levels were combined.

**Fig. 3:**
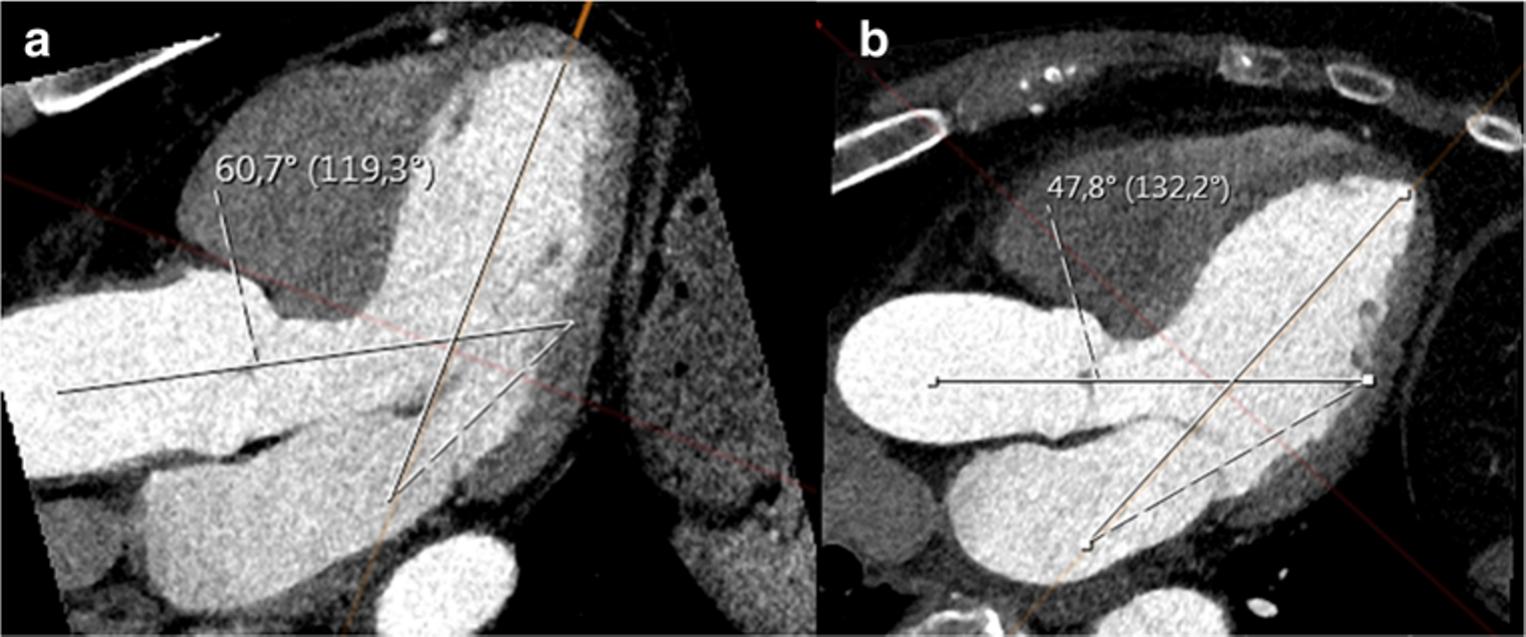
Representative images of the heart-aorta-angle. **a** Dilated ascending aorta (46.8 mm), HAA 119.3°. **b** Normal ascending aorta (36.7 mm); HAA 132.2°

The median HAA was 128.7° (123.3–134.1°) and the mean HAA was 128.8 ± 8.1° in the CCTA patient population. By using the ESC 2014 guidelines, smaller HAA values associated significantly with AA dilatation on all measurement levels: in the sinus valsalva level (*p* < 0.001), in sinotubular junction (*p* = 0.004), and in the mid-AA (*p* < 0.001) in all of the patients analyzed together and in males. In females, smaller HAA values were not significantly associated with AA dilatation (*p* = 0.097–0.507). When all 3 planes were combined, HAA was significantly smaller in the patients with dilated AA (median 126.7°, IQR 121.3–130.8°) compared with the patients with normal AA (median 129.5°, IQR 124.3–135.3°, *p* < 0.001; Table 2 and Fig. 3).

**Table 2:** The association of the heart-aorta-angle (HAA) with ascending aortic dilatation. Normal limits for aorta have been determined by using ESC 2014 guidelines. HAA values are expressed as degrees (°). *ESC*, European Society of Cardiology; *HAA*, heart-aorta-angle. Results are presented as median (interquartile range). Statistical differences have been tested by the Mann-Whitney *U* test

		Sinus valsalva			Sinotubular junction			Mid-ascending aorta			Any plane		
	Gender	Dilated AA	Normal AA	*p* value	Dilated AA	Normal AA	*p* value	Dilated AA	Normal AA	*p* value	Dilated AA	Normal AA	*p* value
HAA (°)	All	126.5 (120.9–130.4)	129.5 (124.3–135.1)	< 0.001	122.7 (117.5–129.9)	128.8 (123.4–134.3)	0.004	125.5 (119.4–131.1)	129.1 (123.7–134.5)	< 0.001	126.7 (121.3–130.8)	129.5 (124.3–135.3)	< 0.001
	Males	125.7 (120.3–130.2)	127.8 (122.6–133.0)	0.003	121.7 (117.9–129.4)	127.0 (121.5–131.7)	0.046	122.2 (118.0–129.3)	127.1 (122.2–131.9)	0.003	125.8 (120.2–130.4)	127.8 (122.6–133.3)	0.003
	Females	128.7 (125.0–130.8)	129.8 (124.6–135.5)	0.097	128.0 (112.9–130.2)	129.6 (124.7–135.3)	0.285	128.7 (123.5–133.7)	129.7 (124.7–135.3)	0.507	128.4 (123.9–133.0)	129.8 (124.7–135.5)	0.114

A smaller HAA correlated very weakly with the body size–adjusted AA diameters (*r* = − 0.256, *p* < 0.001). A smaller HAA associated with AA dilatation also by using age-related formula being 125.5° (118.9–130.9°) in patients with dilated AA and 129.0° (123.7–134.3°) in patients with normal AA, *p* = 0.001, and by using body size–adjusted classification (125.7° [120.2–131.2°] vs. 128.8° [123.6–134.2°], *p* = 0.002).

A smaller HAA associated with AA dilatation by using the ESC classification (*B* = − 3.1°, *p* < 0.001), but not by age-related classification (B = − 1.1°, *p* = 0.374) or body size–adjusted classification (*B* = − 0.8°, *p* = 0.562) in multivariate regression analysis.

**Fig. 4:**
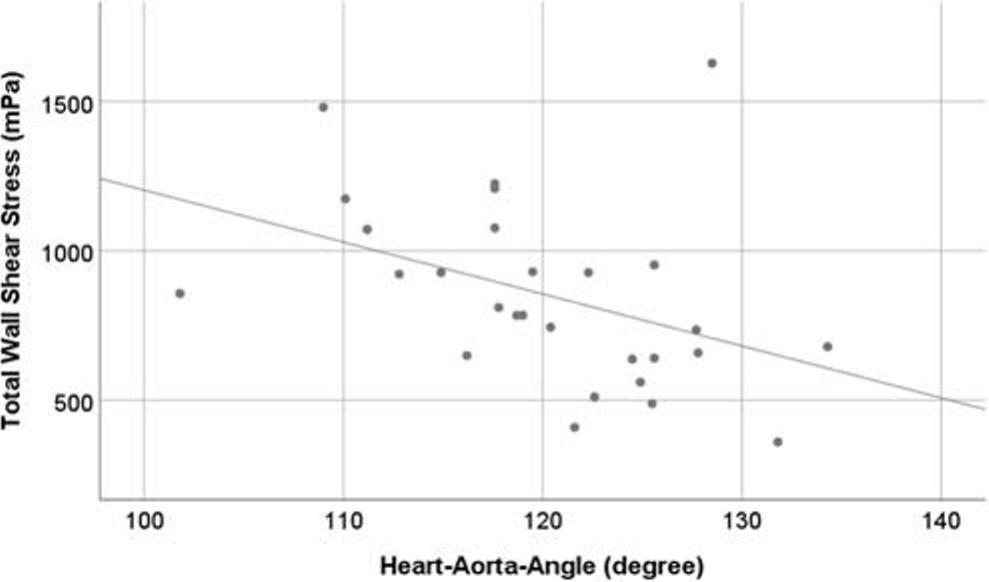
Correlation between the heart-aorta-angle and the total wall shear stress in the proximal part of tubular aorta (*r* = − 0.510, *p* = 0.006) in the outer curvature of aorta in 120° segment

**Fig. 5:**
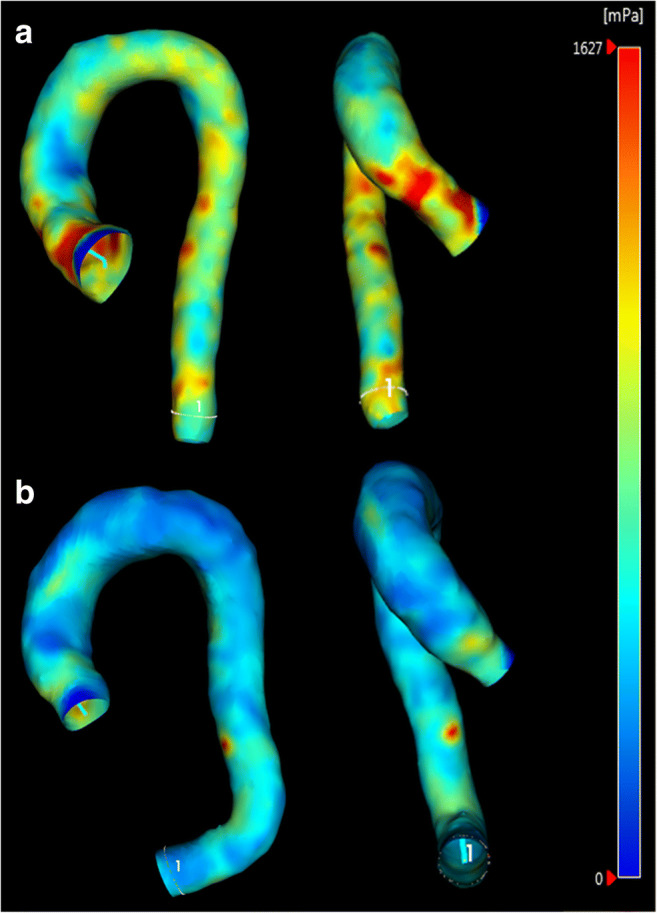
Illustrative wall shear stress (WSS) images of the dilated AA patients. **a** Heart-aorta-angle (HAA) of 118°. WSS is clearly increased in the outer curvature of AA (the red areas). **b** HAA of 132°. WSS is less extensively increased in the outer curvature of AA

When analyzed with 4D flow MRI, smaller HAA correlated significantly with the increased total WSS in the outer curvature of the proximal tubular part of dilated AA (*r* = − 0.510, *p* = 0.006, Fig. 4) in the 120° segment. WSS was not correlated with the smaller HAA in the other planes (*p* = 0.121–0.428). Illustrative 4D flow MRI images of the increased WSS_T_ in the outer curvature of AA in patients with smaller and larger HAA are shown in Fig. 5.

Factors that associated with the smaller HAA are collected in Table 3. Conventional risk factors for cardiovascular diseases such as diabetes (*p* = 0.016), hypertension (*p* = 0.001), hypercholesterolemia (*p* < 0.001), and male gender (*p* < 0.001) and the presence of CAD (*p* = 0.003) significantly associated with smaller HAA. The presence of BAV was also associated with smaller HAA (*p* = 0.025). The smaller HAA correlated weakly with BSA (*r* = 0.213, *p* < 0.001), intraventricular septum thickness (*r* = 0.227, *p* < 0.001), and patient’s age (*r* = 0.191, *p* < 0.001).

**Table 3:** Factors that associated with the heart-aorta-angle in the overall CCTA population. HAA values expressed as degrees (°). *BAV*, bicuspid aortic valve; *CAD*, coronary artery disease; *CCTA*, coronary computed tomographic angiography; *HAA*, heart-aorta-angle. Results are presented as median (interquartile range). Statistical differences have been tested by the Mann-Whitney *U* test. Overweight and obese were determined by using body mass index (≥ 25 kg/m^2^)

	All patients			Males			Females		
	Yes	No	*p* value	Yes	No	*p* value	Yes	No	*p* value
Male gender	126.9 (121.3–131.5)	129.6 (124.7–135.3)	< 0.001	–	–	–	–	–	–
Diabetes	126.5 (121.2–132.6)	128.9 (123.5–134.5)	0.016	126.9 (119.7–133.1)	126.9 (121.4–131.3)	0.722	126.4 (122.4–131.4)	129.9 (124.8–135.5)	0.007
Hypertension	127.9 (122.3–133.1)	129.4 (124.7–135.0)	0.001	126.1 (119.8–130.2)	127.1 (122.4–133.3)	0.014	129.0 (123.3–134.7)	130.4 (126.2–135.6)	0.008
Hypercholesterolemia	127.6 (122.0–133.0)	130.2 (125.1–135.9)	< 0.001	126.1 (119.8–130.1)	127.5 (123.1–133.6)	0.001	128.6 (123.1–133.9)	131.0 (126.7–136.7)	< 0.001
CAD	127.8 (122.3–133.2)	129.3 (124.2–134.9)	0.003	126.7 (119.8–130.9)	127.3 (121.9–131.9)	0.089	129.1 (123.5–134.9)	130.2 (125.1–135.5)	0.149
BAV	126.9 (119.2–130.2)	128.8 (123.4–134.2)	0.025	126.9 (118.7–130.5)	126.9 (121.4–131.6)	0.413	127.1 (118.7–129.9)	129.7 (124.7–135.3)	0.124
Overweight or obese	128.6 (123.1–133.7)	130.8 (125.7–137.6)	< 0.001	126.4 (119.9–130.7)	127.7 (123.3–134.5)	0.023	129.0 (123.4–133.4)	132.5 (126.6–137.8)	< 0.001
Age over the mean	126.9 (121.5–132.7)	129.1 (124.4–134.8)	< 0.001	122.9 (117.6–129.1)	127.2 (121.9–131.9)	0.001	127.9 (122.6–133.5)	130.4 (126.1–135.9)	< 0.001
Intraventricular septum thickness over the mean	127.1 (121.5–133.3)	130.6 (124.9–136.2)	< 0.001	125.2 (120.5–130.1)	127.7 (123.5–133.0)	0.026	128.7 (122.3–134.5)	131.2 (125.3–136.9)	0.004

For further analysis, we selected a subpopulation of 233 patients with no risk factors (diabetes, hypertension, hypercholesterolemia, CAD, BAV, or mechanical aortic valve). The majority i.e. 70.0% of these patients were female and their mean age was 48.3 ± 11.4 years. In this subpopulation, the mean HAA was 131.6 ± 7.5°. According to the ESC guidelines, 35 patients (15.0%) were stratified as having AA dilatation. Furthermore, 39% of males in this subpopulation had AA dilatation. When analyzing the subpopulation with no risk factors, the smaller HAA was still significantly associated with AA dilatation (median 127.9°, IQR 124.3–134.3° in the patients with dilated AA compared with median 131.9°, IQR 127.6–136.9° in the patients with normal AA, *p* = 0.013).

No systematic errors in the HAA measurements were detected between the two independent observers. Intraobserver reproducibility was very strong (ICC = 0.914) and interobserver reproducibility was strong (ICC = 0.870).

## Discussion

This study aimed to evaluate whether the HAA would be associated with AA dilatation. As a result, we found in a consecutive population of 1000 patients that smaller HAA strongly associated with dilated AA. Apart from the structural information in CCTA images, we performed 4D MRI flow analysis in 28 patients with AA dilatation to clarify if this association could be explained by altered flow conditions in AA. It has been earlier demonstrated that in patients with AA dilatation, the aortic flow is displaced into the outer curve of AA leading to increased WSS [10]. In our study, the patients with AA dilatation and smaller HAA showed significant increase in WSS especially in the outer curvature of the proximal aorta. This finding suggests that the smaller HAA leads to more intense angle between the long axis of the heart and proximal ascending aorta, which might have potential to alter the blood flow in AA and, consequently, could lead to increased WSS making the vessel more susceptible to dilatation.

The association between HAA and AA dilation has not been previously evaluated. Aortic elongation occurs with increasing age, which might have an effect on the position of the heart [5]. Smaller HAAs have associated also with AA dilatation by using age-related formula postulated by Hannuksela et al [15]. Furthermore, heart diseases are associated with changes in myocardial morphology which can further associate to HAA. For example, hypertrophic obstructive cardiomyopathy has been shown to be associated with smaller HAA [9]. Increased thickness of intraventricular septum associated with the smaller HAA also in the present study.

Kwon et al presented that the mean HAA was 140 ± 7° in the healthy controls and 128 ± 10° in the hypertensive, elderly patients [9]. In the present study, the mean HAA was 131.6 ± 7.5° in the subgroup of patients with no risk factors. Our smaller values of the HAA in the “healthy” subgroup might be explained that patients had still low-to-moderate pretest probability for CAD; thus, this subgroup cannot be considered as completely healthy patients. The mean HAA was 128.8 ± 8.1° in the overall CCTA patient population, paralleling the results of Kwon et al in hypertensive-elderly patients.

Since it is well known that conventional cardiovascular risk factors, such as hypertension, smoking, diabetes, and hypercholesterolemia, are also risk factors for AA dilatation [17–19], we examined and excluded these risk factors in a selected subpopulation of 233 patients. Even in this subpopulation without these risk factors, the smaller HAA associated significantly with AA dilatation, suggesting that HAA might constitute an independent risk factor for AA dilatation.

Overweight or obesity was associated with smaller HAA in both genders compared to the patients with normal weight. Typically, upper body obesity is more commonly found in males whereas lower body obesity is more commonly found in females [20]. A smaller HAA was strongly associated with male gender. An increased amount of upper abdominal visceral fat especially in males may push the diaphragm and heart to the altered orientation. Although the correlation between BSA and smaller HAA was relatively weak, obesity remains to be one of the few factors to be interfered by lifestyle and medication. However, smaller HAA correlated with AA diameter when it was adjusted to patient’s height and when using the AHI classification method.

The smaller HAA did not reach statistically significant associations with AA dilatation in females. In females, the prevalence of AA dilatation is much lower than that in males when using the ESC dilatation classification [13]. Females have lesser abdominal obesity than males [20]. Further studies are, however, needed to explain why the associations between smaller HAA and AA dilatation are different in males and females.

The main limitations of this study were the higher fraction of female patients and that only a limited volume of AA was included in the image field-of-view. This was a result from the retrospective nature of the study. In addition, a relatively high number of patients had one or more risk factors for cardiovascular diseases and thus, the subpopulation of patients with no risk factors remained relatively small. No follow-up imaging was performed to show causal effects of the smaller HAA on the development of AA dilatation. The presence of obstruction of the left ventricular outflow tract was not registered in CCTA patient population, which might have had an effect on the association between HAA and AA dilatation. However, the presence of the left ventricular outflow tract obstruction is estimated to be very low in this relatively healthy population. Furthermore, the patients in 4D MRI flow study were older and mostly male which limits the comparison with the CCTA patients.

The HAA measurement method, used in this study, has shown to have high intraobserver and interobserver reproducibility in earlier studies [9]. Our study supports the previous findings of high reproducibility of HAA measurements. Since the measurement of HAA is straightforward, fast, and reproducible, it provides an additional tool to analyze CT images of the aorta and heart. The measurement may have a potential as an additional indicator predicting further AA dilatation and stratifying patients for follow-up CT examinations, together with other risk factors such as BAV or genetic risk factors for AA dilatation.

To conclude, the smaller HAA associates significantly with AA dilatation. The smaller HAA increases WSS in the outer curvature of the proximal AA. The clinical relevance of HAA needs to be further verified especially the contributions from the upper abdominal visceral fat and aortic elongation. Thus, further clinical and imaging follow-up studies are needed to verify the possible clinical value of this index.

## Abbreviations

AA: Ascending aorta
AHI: Aortic height index
BAV: Bicuspid aortic valve
BSA: Body surface area
CAD: Coronary artery disease
CCTA: Coronary artery computed tomography angiography
CT: Computed tomography
ESC: European Society of Cardiology
HAA: Heart-aorta-angle
ICC: Intraclass correlation coefficients
IQR: Interquartile range
LV: Left ventricle
MRI: Magnetic resonance imaging
TRUFI: True fast imaging with steady-state precession
WSS: Wall shear stress
